# Computational Modulation of the V3 Region of Glycoprotein gp125 of HIV-2

**DOI:** 10.3390/ijms22041948

**Published:** 2021-02-16

**Authors:** Patrícia A. Serra, Nuno Taveira, Rita C. Guedes

**Affiliations:** 1 Department of Pharmaceutical Sciences and Medicines and Research Institute for Medicines (iMed.ULisboa), Faculty of Pharmacy, Universidade de Lisboa, Avenida Professor Gama Pinto, 1649-003 Lisboa, Portugal; pfserra@ff.ulisboa.pt; 2 Centro de Investigação Interdisciplinar Egas Moniz (CiiEM), Instituto Universitário Egas Moniz, Monte de Caparica, 2829-511 Caparica, Portugal

**Keywords:** HIV, viral glycoprotein, homology modeling, molecular dynamics, structural elucidation, structure-function relationship

## Abstract

HIV-2 infection is frequently neglected in HIV/AIDS campaigns. However, a special emphasis must be given to HIV-2 as an untreated infection that also leads to AIDS and death, and for which the efficacy of most available drugs is limited against HIV-2. HIV envelope glycoproteins mediate binding to the receptor CD4 and co-receptors at the surface of the target cell, enabling fusion with the cell membrane and viral entry. Here, we developed and optimized a computer-assisted drug design approach of an important HIV-2 glycoprotein that allows us to explore and gain further insights at the molecular level into protein structures and interactions crucial for the inhibition of HIV-2 cell entry. The 3D structure of a key HIV-2ROD gp125 region was generated by a homology modeling campaign. To disclose the importance of the main structural features and compare them with experimental results, 3D-models of six mutants were also generated. These mutations revealed the selective impact on the behavior of the protein. Furthermore, molecular dynamics simulations were performed to optimize the models, and the dynamic behavior was tackled to account for structure flexibility and interactions network formation. Structurally, the mutations studied lead to a loss of aromatic features, which is very important for the establishment of π-π interactions and could induce a structural preference by a specific coreceptor. These new insights into the structure-function relationship of HIV-2 gp125 V3 and surrounding regions will help in the design of better models and the design of new small molecules capable to inhibit the attachment and binding of HIV with host cells.

## 1. Introduction

AIDS (acquired immune deficiency syndrome) was discovered more than forty years ago, however, until now there is still no vaccine or cure for this disease. AIDS is caused by two human immunodeficiency virus (HIV) types, HIV-1 and HIV-2. HIV-1 is pandemic while HIV-2 is mainly confined to West Africa and western European countries (e.g., France, and Portugal). In areas where HIV-2 is endemic, co-infection with HIV-1 is very common [1]. Like HIV-1, HIV-2 is transmitted through direct contact with HIV-infected body fluids (blood, semen, and vaginal fluids) or from mother to newborns during pregnancy, delivery, or breastfeeding [2]. Aside from a similar mode of transmission, these two viruses share similar intracellular replication mechanisms and clinical consequences. In fact, as the disease progresses, people infected with HIV-2 will become vulnerable to the same spectrum of associated opportunistic infections and co-morbidities as people infected with HIV-1 [3]. The HIV-2 infection is frequently neglected in global HIV/AIDS campaigns. However, if we want to eradicate AIDS by 2030 [4], a special emphasis must be given to HIV-2, which poses distinct challenges for prevention, diagnosis, and treatment. Distinct from HIV-1, HIV-2 is less virulent and only a small percentage of HIV-2 infected patients progress to AIDS. Disease progression is much lower relative to HIV-1 infected patients, leading to a high percentage of asymptomatic patients, lower transmission rate, and longer latency, resulting in a longer disease-free survival [5]. The mechanisms that enable the human host to better control the HIV-2 infection by itself are far from being completely understood. The evolution of CD4+ T-cell counts declining is slower when compared with HIV-1 infection [6,7,8] and HIV-2 is highly susceptible to antibody neutralization [7]. A better understanding of the immunopathogenesis of HIV-2 infection could lead to the production of new weapons against HIV [6,9].

Still, as mentioned above, infection with HIV-2 leads to AIDS in most patients and effective antiretroviral treatments are crucial for preventing disease progression and cure [10]. Contrary to HIV-1 infected individuals, most HIV-2 patients in a chronic stage of the disease produce and preserve potent neutralizing antibodies [11]. This may be linked to specific conformations of envelope proteins on the surface of the virus. Recently, virus isolates from patients in advanced stages of infection proved to be more resistant to entry inhibitors, including the CCR5-antagonist maraviroc, than isolates from asymptomatic patients [12]. However, with disease progression, CD4+ T-cell counts in HIV-2 infected individuals tend to become similar to HIV-1 as the immunological differences tend to disappear and the mortality risk becomes equivalent [6,13]. Likewise, the susceptibility to antiretroviral drugs and drug resistance development is distinct between HIV-1 and HIV-2 [8,14].

To enter the host cell, HIV surface envelope glycoprotein first binds to the CD4 receptor and then to the CCR5 and/or CXCR4 coreceptor through the V3 loop region. The conformational change that follows binding to the co-receptor releases the hydrophobic fusion peptide at the amino-terminus of the transmembrane gp41 envelope which is crucial for the subsequent virus-to-cell fusion [15]. The viral entry mechanism, and in particular, the glycoproteins positioned at the surface of the virus envelope, are good targets for the development of new therapeutics [8,16,17,18]. Until now, only two therapeutic agents targeting the viral entry pathway successfully reach the market: maraviroc [19], a small molecule CCR5 antagonist, and enfuvirtide, a peptide derived from the helical region 2 in the ectodomain of gp41 that binds to the helical region 1 and prevents the six-helical bundle formation that precedes virus-to-cell fusion [18]. Understanding the structural basis of interactions between the HIV envelope proteins and their human host cells are of major importance for the identification of new drugs that prevent HIV entry. However, very few studies have investigated the HIV-2 cell entry process, and this is mainly due to the lack of experimental structural information of the envelope glycoproteins.

Computational methods have been successfully applied to predict reliable 3D protein structures that will lead to a deeper understanding of its behavior and function, protein-ligand interactions, and of the impact specific mutations, insertions, and deletions have on its conformation and hence on its function [20,21,22,23].

**Figure 1:**
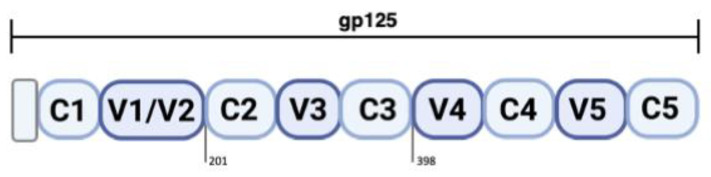
Graphical representation of the C2V3C3 domain. The HIV-2 gp125 is divided into five hypervariable regions, named V1 to V5, delimited by five conserved regions, named C1 to C5.

Here, we modeled the C2V3C3 domain of HIV-2 surface glycoprotein gp125 comprising the variable region V3 to explore and gain further insights at the molecular level into protein structure and interactions crucial for HIV-2 entry. The protocol carried out was based on the generation and optimization of the 3D structures of the HIV-2 env gp125, wild-type, and mutant species, using homology modeling (HM), focusing on the C2V3C3 domain (Figure 1). The predicted structures were validated to check the quality of the models. V3 is a key functional loop of the HIV-2 envelope and plays an important role to determine co-receptor specificity, use, cellular tropism, and antibody neutralization [24]. The conserved regions C2 and C3 are used to support the V3 region (Figure 1).

Important mutant species were also modeled. We know from the literature that a single substitution of lysine by histidine on position 18 in V3 can be used to predict CXCR4 usage in HIV-2ROD (Human immunodeficiency virus type 2, ROD strain, was the first reported isolate of HIV-2, which originated from the Cape Verde Islands, Senegal). H18L mutation was sufficient for a full X4-to-R5 tropism switch in the context of the short version of the V3 loop (ΔH23Y24) [25]. Furthermore, the modification of specific residues in V3 resulted in an easier neutralization when compared with the wild-type as well as an alteration of the capacity of replication in different cell types (manuscript in preparation). To assess the importance of these residues at positions 18, 23, and 24 of V3, the wild-type HIV-2ROD gp125 V3 loop model was analyzed along with six other models incorporating selected mutations and/or deletions. Molecular dynamics simulations (MD) were performed to optimize the models, and the dynamic conformational behavior was taken into account for structure flexibility and interactions network formation.

## 2. Results

Current research efforts are mainly focused on and directed to HIV-1 due to its higher spreading, virulence, and transmissibility. Thus, HIV-2 has been neglected due to its endemic nature, limited access to patients, and associated scarcity of data. However, a broader knowledge of HIV-2 can be valuable to provide insight into the immune response against HIV-1 [26]. The first and only crystallographic three-dimensional structure of the gp125 of HIV-2 dates back to 2016 [27]. The limitation of this structure was the missing variable domains. V3 was partially resolved, presenting the truncated residues at the limits of the domain. Even though the identity between the glycoproteins of HIV-2 and HIV-1 was lower compared to HIV-2 and SIV, the structure resolved was identical to the HIV-1 gp120 core, adopting similar conformations. The analysis and conclusions afforded by the crystal structure of the glycoprotein of HIV-2ST supported the need to further study the structure of the variable domains of the virus [27].

**Figure 2:**
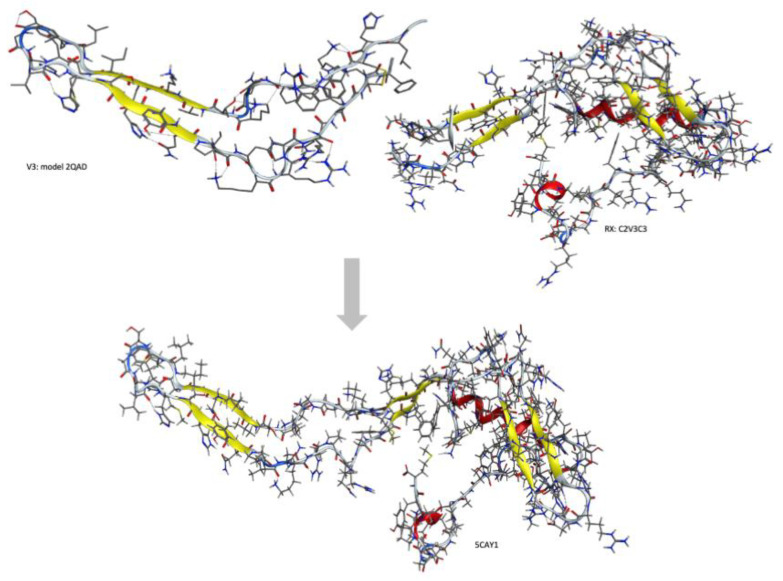
Graphical representation of the domains merged in the new model of the C2V3C3 domain.

To obtain the 3D structures of HIV-2ROD wild-type and mutant isolates, a homology modeling campaign was implemented combining the analogous gp120 glycoprotein of HIV-1 and the partial structure of the HIV-2 gp125, using the Molecular Operating Environment program (MOE) version 2015.10 (http://www.chemcomp.com/software/) [28]. Structural models of the C2V3C3 region of gp125 of HIV-2ROD were built by homology modeling using the crystal structure of 5CAY as the main template (HIV-2), combined with the structure 2QAD (HIV-1) (Figure 2).

In addition, six models of six C2V3C3 loop variants resulting from combinations of mutations and/or deletions of specific residues were also constructed (Table 1). The models were then subjected to MD simulations to account for structural flexibility and optimization. The region of interest, C2V3C3, was established as previously reported by Barroso and co-workers [29]. The HIV-1 and HIV-2 sequences are different in terms of the number of residues that define gp120 and gp125 (Table 2). The definition of the V3 variable domain was equal for both viruses as V3 was defined by a cysteine bridge between the first and last cysteine of the V3 region.

### 2.1. Modeling the Structure of HIV-2 V3 Loop

The combination of the HIV-2 available crystallographic arrangement with the HIV-1 V3 crystal model derived from the 2QAD structure was the most promising for the construction of the final model of the HIV-2 gp125 C2V3C3 domain. The selected templates of the glycoprotein were complexed with CD4 leading to the preservation of the binding conformation. The template selection was based on the fact that the V3 region has a similar structure and functions in both HIV-1 and HIV-2 [27].

The HIV-1 structures 2QAD [30] and 2B4C [31] preserved most of the V3 residues among all of them and presented, as well, the higher value of identity and similarity. As mentioned above, the only available crystal structure of gp125 was devoid of variable regions. We, therefore, combined the partial crystal structure of gp125 of HIV-2ST strain (5CAY) with the variable domains from HIV-1 crystal templates [27]. In order to select the best model to merge with the crystallographic structure, all available HIV-1 models were superimposed and aligned to 5CAY.

The alignment of the HIV-2ROD strain (sequence of interest) and HIV-2ST strain (5CAY) led to a high percentage of identity and similarity between them: identity 86% to full-length gp125; similarity 93% to full-length gp125. With this result, we were able to continue the generation of the model. The final combination of HIV-2ST 5CAY and HIV-1YU2 2QAD crystal structures was selected to construct the final model. Table 2 presents the V3 sequence of the structures used in model creation using the sequence of HIV-2ROD as the reference and the sequence of V3 to modulate.

In order to validate the models, it was necessary to evaluate the compliance with quality factors. The Ramachandran plots results of the homology models created with MOE software package and analysis of the number of outliers of each model was performed and confirmed that the V3 were mainly localized in energetically favorable regions of the Ramachandran plot as well as their dihedral χ1 angles (Supplementary Information 1). The validation of the ROD wild-type (WT) model using the normalized z-DOPE score [32] was 0.455 and GA341 score [33] was 0.999. Using ERRAT software the model score was 90.38%. The conjugation of all the validation data allows the model to be used in further studies (Supplementary Information 1).

Since the information about the structural characteristics of the variable domains was reduced, a secondary structural prediction was a useful tool to analyze the constructed models. The secondary structure of constructed models was compared with structural predictions performed using Robetta server [34] (http://robetta.bakerlab.org/) and PSIPRED [35] (http://bioinf.cs.ucl.ac.uk/psipred/) software. All the models took into account the spatial constriction imposed by the presence of a disulfide bridge between cysteine at position 1 and cysteine at position 36 of V3.

Table 3 presents the similarity between the results of the structural predictions and the final model selected. A prominent presence of β sheet structural definition was visible, which supports the fact that the V3 domain was a loop with high organized mobility supported by this structural definition

### 2.2. Structural Analysis of WT and Mutated HIV-2 V3 Loop

**Figure 3:**
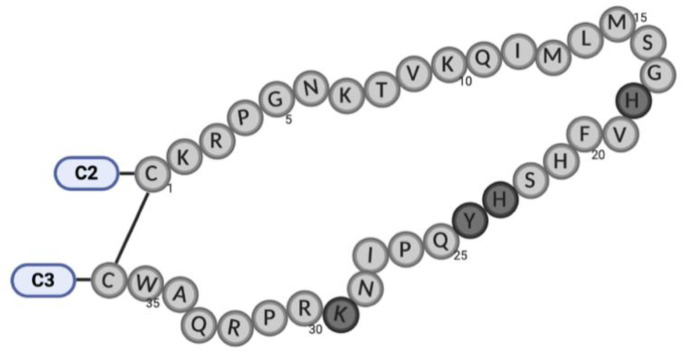
Graphical representation of the HIV-2ROD wild-type (WT) V3 domain. The amino acids 18, 23, 24, and 29 highlighted in dark grey were the ones modified in the mutated species.

**Figure 4:**
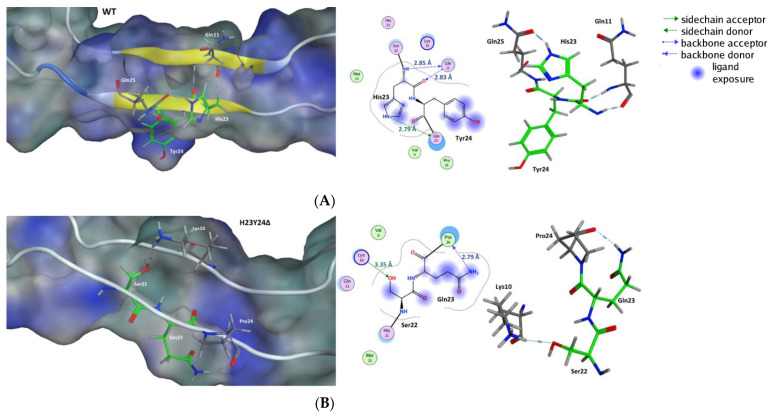
Potential structural impact of the deletions H23 and H24 in the V3 loop of HIV-2 gp125. The impact of the deletions ΔH23Y24 in the V3 region is very evident in the modification of the secondary structure and in the total modification of the interactions network associated with this region and due to Ser22 and Gln23 being now covalently bonded. The residues in green are directly involved in the mutations. The structures (**A**,**B**) present a surface in a variable range of color of teal blue for a hydrophilic influence on a strong blue for a lipophilic influence.

To identify specific structural features of V3 (Figure 3) correlated with its function in the virus replication cycle, three-dimensional (3D) structures of the reference isolate HIV-2ROD (name wild-type (WT)) and several mutant V3 loops (Table 1) were generated by homology modeling. In our models, the structure of the V3 loop of ROD WT was characterized by a 23% β sheet and a null percentage of α helix (Table 3). His23 and Tyr24 amino acids fit well on the β sheets of ROD WT V3. His23 interacted with glutamine at positions 11 and 25 (Figure 4A). Tyr24 could establish π-π interactions due to the clear exposure of the aromatic system to the environment. In addition, the exposure of the hydroxyl group in the aromatic system of Tyr24 may promote additional interactions with the environment. The deletion of these residues resulted in a significant loss of the aromatic system and led to the elimination of the parallel β sheets present in the V3 loop. The loss of aromaticity may possibly interfere with the hydrophobic equilibrium and influence receptor and co-receptor binding [36]. Deletion of these two residues led to a new set of interactions between serine at position 22 and lysine at position 10, and between glutamine at position 23 and proline at position 24 (Figure 4).

**Figure 5:**
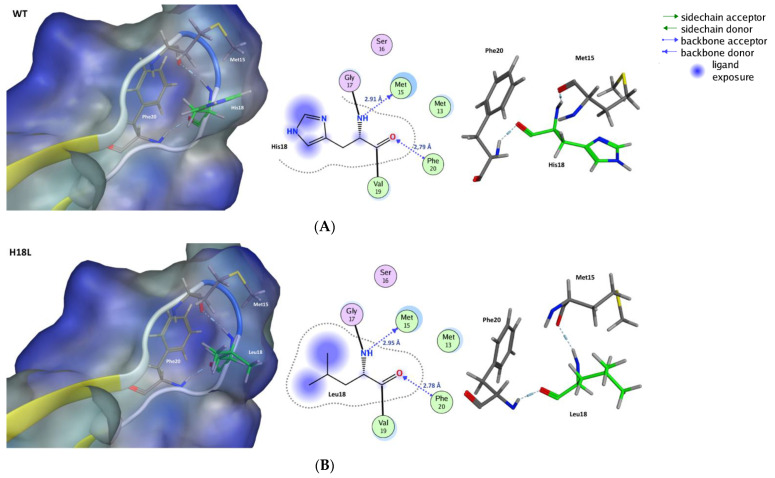
Potential structural impact of the H18L substitution in the V3 loop of HIV-2. Due to the interactions being performed using the atom of the backbone, the hydrogen bonds formed with Leu18 are conserved as observed in the WT His18 site. The residues in green are directly involved in the mutation. The structures (**A**,**B**) present a surface in a variable range of color of teal blue for a hydrophilic influence to a strong blue for a lipophilic influence.

The binding of gp125 glycoprotein with the cell receptor CD4 led to conformational changes and subsequent interaction with the co-receptor. The aromatic system at position 18 (His18) in V3 might influence the specificity of interaction of gp125 with the co-receptor [36]. His18 formed H-bonds with methionine at position 15 and phenylalanine at position 20. These interactions appeared to promote the definition of the tip of the loop (Figure 5A). The aromatic moiety of histidine conferred a versatile range of molecular interactions: hydrogen bond donor and acceptor, the ability of acid ionization, and acted as the coordinating group in several mechanisms involving metallic cations [37]. The H18L variant replaced a basic residue with a hydrophobic residue and leads to the loss of the aromatic moiety which abrogated any possibility of establishment of π-π interactions within the V3 environment. Leucine18 formed H-bonds with Pro24 and Met15, promoting the organization of the structure of the tip (Figure 5).

**Figure 6:**
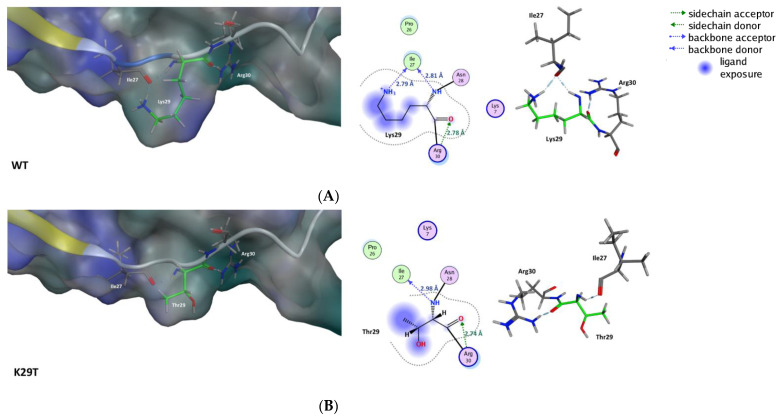
Potential structural impact of the K29T substitution in the V3 loop of HIV-2. The residues that participate in the interactions in the neighborhood are preserved with the mutation. The residues in green are directly involved in the mutation. The structures (**A**,**B**) present a surface in a variable range of color of teal blue for a hydrophilic influence to a strong blue for a lipophilic influence.

The substitution of lysine at position 29 (K29) by a threonine reduced the charge of V3 and led to the partial loss of the interactions with isoleucine at position 27. Modification of the side chain due to mutation of residue 29 abolished one of the H-bond formed with Ile27 (Figure 6B, Table 4). The mutation K29T caused a reduction in the exposition to the environment in this region (Figure 6B, Table 4) and the modification of the charge of this domain. Besides this, no other significant deviations were identified. This suggests a similar behavior compared with the wild-type.

**Figure 7:**
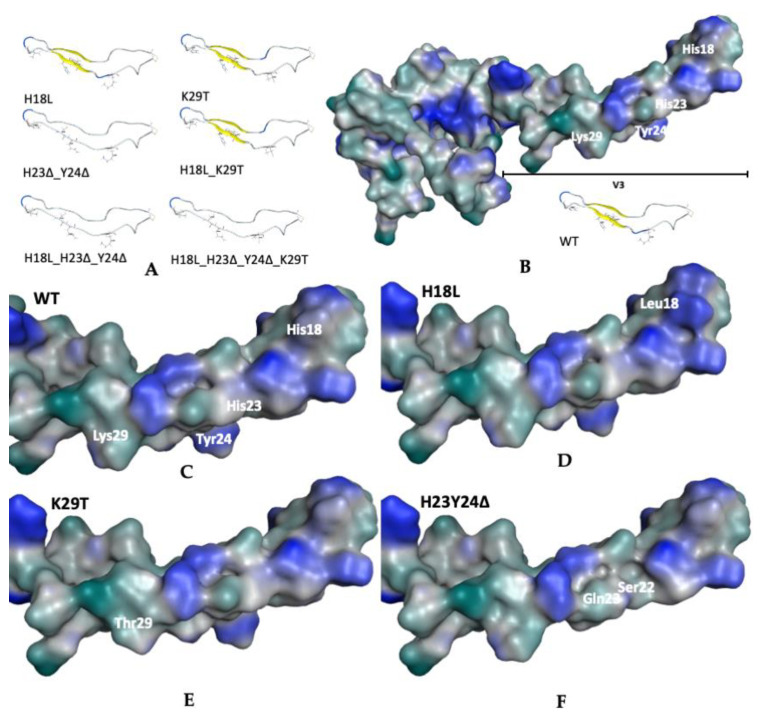
Visual representations of molecular surfaces of the wild-type and the potential impact of the variants analyzed. (**A**) Three-dimensional structure of the secondary structural definition of ROD WT and mutant V3 loops determined by homology modeling. (**B**) Surface model of the full C2V3C3 domain and the V3 variants WT (**C**) and the mutants H18L (**D**), K29T (**E**), and ΔH23Y24 (**F**). The structures present a surface in a variable range of color of teal blue for a hydrophilic influence on a strong blue for a lipophilic influence. It is highly evident the exposition of the residue Tyr24 and His18, associated with a major lipophilic contribution.

Calculations of the solvent-accessible surface area (SASA), using MOE, showed mostly a decrease of SASA of the mutants when compared with ROD WT, except for H18L (Table 4). No significant deviations were observed on the length of the V3 loop with the performed mutations, however, a substantial increase in the width of the V3 loop was detected particularly for the structures containing the H23 and Y24 deletions (Figure 7). The deletions of the residues at positions 23 and 24 led to the loss of the H-bond between the stems of the loop, which can explain this variation.

### 2.3. Molecular Dynamics

**Figure 8:**
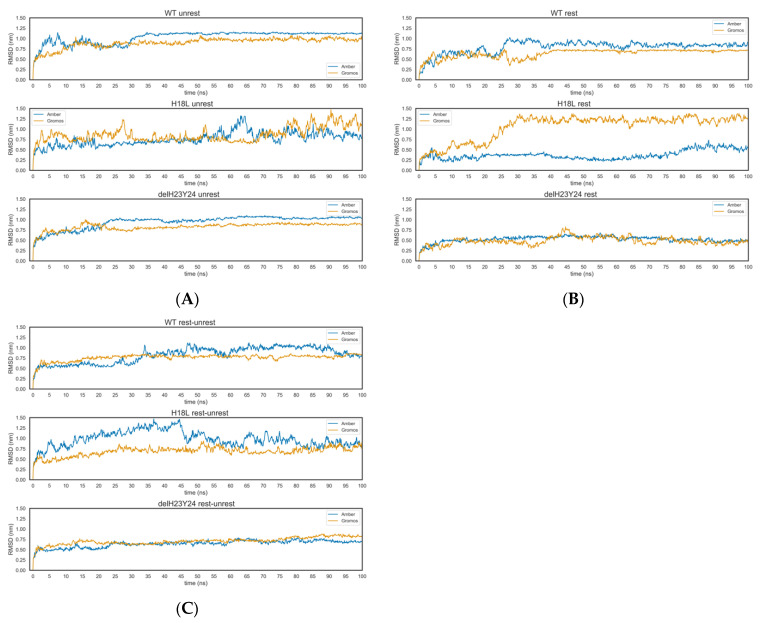
RMSD plots of the systems used for molecular dynamics. (**A**) RMSD plots for the unrestricted system; (**B**) RMSD plots for the restricted system; (**C**) RMSD plots for the unrestricted after restricted system.

**Figure 9:**
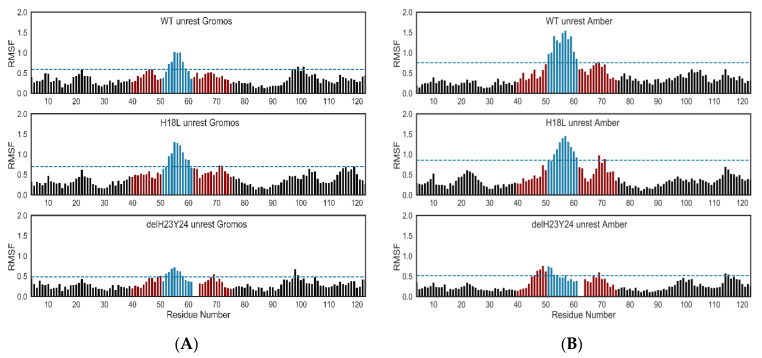
RMSF plots of the unrestricted systems. The blue range corresponds to the set of the most variable residues along the simulation. The dark red range of residues defines the V3 domain and the black bars define the C2 and C3 domain. Most variable residues were situated at the tip of the V3 and the residue at position 18 is very near this most changeable area through simulation. The plot numerates the residues starting in 1 at the first residue of C2. To identity the V3 residues the original numeration is between brackets in the text. (**A**) RMSF for the systems using Gromos 54a7 force field; (**B**) RMSF for the systems using Amber 99SB-ILDN force field.

HIV glycoproteins are enrolled in a dynamic entry mechanism where variable domains are highly mobile. These structures undergo structural rearrangements in order to interact with receptors and co-receptors enabling fusion with the membrane and internalization of the viral content into the cell [38]. Therefore, and in order to relax the C2V3C3 structures generated by homology modeling and to address how the mutations may affect the structural reshaping of this variable domain, a 100 ns MD simulation was performed. All molecular simulations were conducted using the Gromacs package. Starting coordinates were taken from previously generated homology models. Each system was independently simulated using two force fields (Amber 99SB-ILDN and Gromos 54a7). Three different states of restrictions were used in independent runs: 1. C2 and C3 restricted along the simulations (named restricted); 2. C2 and C3 unrestricted along the simulations (unrestricted); 3. C2 and C3 unrestricted after a restricted simulation (rest-unrestricted). All systems used are enumerated in Table 5. The fluctuation in potential energy, the RMSD (root-mean square deviation) of Cα atoms, and RMSF (root-mean square fluctuation) from the structures were monitored during the MD simulation time (Figure 8 and Figure 9). The potential energy of all systems was stable along the simulation period (Table 6). The RMSD values of Cα atoms were calculated after fitting the Cα atoms to the initial structure. Overall, the RMSD values converged after a 40-ns simulation (Figure 8, Table 7).

To assess the structural flexibility and gain insight into the effect of structural modifications in functional differences, the H18L and ΔH23Y24 models were subjected to MD simulation under the same protocol and using both force fields. The simulations made evident the reinforcement of the definition of the secondary structure already present in each system. No significant differences were found for WT and H18L systems. The RMSD results were more variable when comparing the same systems in different restrictions. However, as mentioned previously, the mutated systems and WT system tended to stabilize after the 40 ns. Using the Gromos 54a7 force field wild-type models reached the lowest values of RMSD. Even using the Amber 99SB-ILDN force field, the curve was less variable along the time and the RMSD values were higher. The mutated systems showed a more divergent behavior in terms of stability (Figure 8).

The molecular dynamics allowed a detailed analysis of the mutated residues. The use of two force fields, Amber 99SB-ILDN and Gromos 54a7, and different restrictions of the C2 and C3 domains revealed different behaviors. The H18L mutant moved farther from the standard profile of the WT and ΔH23Y24 variant. Focusing on the unrestricted systems, both variants stabilized the RMSF around 1.00 nm, a smaller value to the Gromos 54a7 force field. The H18L mutant presented a more variable RMSF between the 0.75 and 1.50 nm along all simulations. The same pattern was observed in the restricted-unrestricted system (Figure 9). The variable domain was characterized by a high degree of mobility and flexibility. In terms of fluctuation of the atomic positions of the residues, it was evident that the tip of V3 is enrolled in the most changeable region. In almost all systems the region with a higher RMSF was defined between the residues 12 to 22 (51 to 61), in which the muted residue 18 (57) is located. The H18L mutated systems had the most variable residues along the time.

To understand how molecular interactions fluctuate with different mutations and how this can impact protein function, all the intra-molecular interactions established in the specific structures studied were investigated and then compared. The Protein Ligand Interaction Profiler (PLIP) tool was chosen to evaluate the molecular interaction in defined positions of the protein. A special focus was placed upon the mutated positions of the V3 domain. To this end, the protein files were formatted in order for the software to consider the residue/position under study as a ligand. This analysis enabled the characterization of the most prevalent pattern of interactions between key residues of a given protein system along the simulation. The plots for each system can be found in Supplementary Information 2.

## 3. Discussion

The lack of a complete crystallographic structure limited the access to structural information using the gp125 of HIV-2. The high flexibility and variability associated with this structure reinforce the importance of the structural selection. Barroso et al. generated a model of HIV-1 C2V3C3 domain and HIV-2 C2V3C3 domain, using HIV-1 JR-FL gp120 (PDBID: 2B4C) and an unliganded SIV gp120 envelope glycoprotein (PDBID: 2BF1), respectively as templates [12,29]. The selected X-ray structures (templates) differ in the number of V3 amino acids. Both factors, namely the absence of CD4 structure and the different number of amino acids present in the templates, influence the generation of the homology model. Concerning the absence of CD4 in one of the templates used, it is important to notice that to initiate the viral entry process into the cell, a closed assembled envelope trimer binds to a single CD4 monomer, stabilizing the intermediate state of the envelope [39]. Additional CD4 monomers bind to this intermediate and lead to the formation and exposure of the gp125 (for HIV-2) co-receptor binding site. Unlike HIV-1, HIV-2 can use multiple co-receptors besides CCR5 and CXCR4 and can even use these coreceptors without binding to CD4 [40]. This implies higher structural flexibility of the regions in gp125 that are thought to mediate binding to the coreceptors, V3, and eventually V1 and V2 regions [12]. The binding to the co-receptor lead to conformational modifications, focused mainly on the transmembrane unit, responsible for the fusion of the virus and host membrane [39]. Taking the viral entry mechanism into account, the use of structures crystalized in different stages of the process can alter and bias the 3D structure of the model to acquire a specific conformation. In the construction of the present model to improve the definition of the C2V3C3 domain, the sequence of HIV-2ROD was aligned with the three selected templates (Table 2). The combination of the HIV-2 available crystallographic structure with the 2QAD structure was the most promising to the construction of the model. These structures were crystalized using the glycoprotein complexed with CD4 leading to the preservation of the binding conformation and standardizing the conformation of all the compared structures.

Structurally, the mutation H18L leads to a loss of an aromatic moiety, very important for the establishment of π-π interactions, which could induce a structural preference by a specific co-receptor. Modifications on the aromatic residues at positions 18 and at positions 23 and 24 of V3 suggest an important feature to determine co-receptor usage. Indeed, it was seen that modifications at these positions in different variants originate an increment in the CCR5 usage and, if these two mutations occur in the same variant, there was a specificity to co-receptor CCR5 [12,25]. In this variant, there was a significant alteration on the aromatic system, suggesting that the presence of aromatic systems favors CXCR4 usage.

Histidine at position 23 was present in two parallel hydrogen bonds with glutamine at position 11 which could be essential to promote the spatial arrangement of the loop, maintain both β sheets, and possibly promote the interaction with coreceptor CXCR4. Consistent with this, variant ΔH23Y24 V3 loop, which binds to CCR5 instead of CXCR4, presented a major variation of width and solvent-accessible surface area. Results also showed a major distance of the hydrogen bond between histidine at position 18 and phenylalanine at position 20 on variant ΔH23Y24. This is consistent with the importance of position 18 for coreceptor binding.

### Molecular Interactions in the MD Runs

The protein-ligand interactions are of utmost value to gain information into how two entities interact between them [41]. The arrangement of a protein is dependent on the non-covalent interactions promoted with itself, the environment, and its ligand [42]. The detailed description of the set of interactions in a system is crucial to a deeper understanding of molecular recognition and protein function. The PLIP web service is an open-source software enabling the detection and visualization of protein-ligand interaction patterns [42]. The heatmaps of protein interactions numerate the residues starting in 1 at the first residue of C2. To identity the V3 residues in the plot, the original numeration is between brackets.

The mutated systems and WT tend to stabilize after the 40 ns, as reported in the RMSD plots. Analyzing the PLIF plots, the systems showed similar behavior. The interactions tend to differ at the second part of the trajectory. There are permanent interactions along the whole trajectory, and they are common to the systems. During the simulation, the most prevalent residues involved in atomic interactions between the His18 (57) and the protein are Met15 (54), Val19 (58), and Phe20 (59). After 70 ns, more conservation of the existent interactions is evident, using the Amber 99SB-ILDN force field. In the systems using Gromos 54a7 force field, it stabilizes sooner, as already seen in the RMSD analysis. The most prevalent residues are in concordance with the interactions already reported for the homology modeling structures. In the case of the restricted systems, there was a lower variation of interactions along the simulation, confirming the behavior reported in the RMSD analysis.

The Histidine at position 18 is enrolled mostly in the H-bond interaction type. In the unrestricted system of Amber 99SB-ILDN force field, the Leu14 (53) at V3, and Asn24 and Asn106 at C2 and C3 formed H-bonds with the His18, suggesting a closer proximity of the V3 region to the conserved domains after the 60 ns of simulation. Analyzing the systems of the Gromos 54a7 force field, the interactions registered along the simulation are formed with neighbor residues of V3. Interaction of His18 with residues Met15 (54), Val19 (58), and Phe20 (59) is constant along the simulation. The His21 (60) is enrolled in the first 18 ns of the simulation, with interactions with Ser16 (55) constant until the 70 ns of simulation and interactions with Gly17 (56) intermittent along the simulation, which is similar in both force fields. The restricted systems are distinct. A minor variation in the interactions formed along the simulation is visible.

The residue at position 18 is located at the end of the descending β sheet. The hydrogen bond interactions between His23 carbonyl oxygen and Gln11 (2.11 Å) and between His23 (NH) and Gln25 (carbonyl oxygen) promote a regular secondary structure motif, as observed in Figure 4. The assessment of Figure 4A (WT) and 4B (ΔH23Y24 mutant) reveals the loss of this interaction pattern and consequently the loss of secondary structure. In the homology modeling systems, the mutants with deletions lost this specific motif resulting in a major difference in the interactions promoted by the His18. The unrest ΔH23Y24 system, until the 70 ns, is characterized by a higher variance in the formation of interactions. His18 contacts with the residues that form the lateral side of the V3, from histidine right before the cysteine that begins the V3 until Ser16. The disorganization associated with the loss of the β sheet is quite evident in this interaction pattern. The Val19 (58) and Phe20 (59) are involved in interactions, as seen in the other systems. Additionally, the Ser16 (55) forms a stable interaction after the 65 ns. The systems of the Gromos 54a7 force field reproduce the interactions with the residues on the lateral side of the V3. This fact indicates the collapse of the tip into the V3 associated with the high mobility of the domain. The residues Val19 (58) and Phe20 (59) interact with His18 along all simulation, and with Met15 (54) until 70 ns.

Mutation H18L removes the aromatic moiety at this position, introducing a nonlinear aliphatic chain and modifying the chemical environment of this site.

The residues involved in the major interactions with Leu18 are Met15 (54), Ser16 (55), Val19 (58), and Phe20 (59) after 50 ns. Sporadically, at the first 50 ns of simulation, hydrophobic contacts are registered with residues of C2 and C3 domains. In the Gromos 54a7 force field systems, there were no contacts with the C2 and C3 domains. Additionally, Leu14 (53) presents a hydrophobic contact with Leu18 after 35 ns. The rest-unrestricted systems present a similar behavior as described for the unrestricted systems for the mutation H18L. The restricted systems showed similar behavior at the Amber 99SB-ILDN force field. In the Gromos 54a7 force field, the interactions formed are more constant along the simulation.

Lysine 29 is located at the end of V3, in a turn that precedes the area where this domain bends. In the experimental results, the K29L V3 mutant was the only one that kept the affinity for the CXCR4 coreceptor [25]. The contacts formed by Lys29 are more variable along the systems. The Asn28 (67), Arg30 (69), and Arg32 (71) form H-bonds along the WT unrested simulation, in particular Arg30 (69) that presents a constant interaction along the 100 ns, consistent with the model created with homology modeling. Asn28 (67) starts to interact with Lys29 after 15 ns. The presence of the interaction with Arg32 (71) is more fluctuant. Until 30 ns, hydrophobic contact between Arg31 (70) and Lys29 formed transiently, being substituted by a hydrogen bond with more frequency. The other residues that interact with K29 are less frequent. This behavior is very similar to the unrestricted system of ΔH23Y24 of Gromos 54a7 force field. In the WT system of Gromos 54a7 force field, Asn28 (67), Arg30 (69), and Arg32 (71) form interactions frequently, being more variable along the simulation. Frequently, Pro31 (70) forms contacts with K29. Gln11 (50) and Glu 119 (C3) formed H-bonds with this residue after 45 ns. In the H18L system of Amber 99SB-ILDN force field, Arg30 (69) interacts with Lys29 during all simulations. The discontinued interactions are registered, mainly, after 40 ns. Interaction with Ile27 (66) is present in the structure of homology modeling and in this system Ile27 (66) forms H-bond after 40 ns. In the H18L system of the Gromos 54a7 force field, the interaction profile is different. Ile27 (66), Asn28 (67), Arg30 (69), and Arg32 (71) contact K29 throughout almost all simulations. Pro04 interacts hydrophobically with Lys29 and interruptedly with the residues that initiate the V3 domain, corresponding to the parallel side of this residue. In the ΔH23Y24 system of Amber 99SB-ILDN force field, the interactions formed with K29 are with the residues in the neighborhood, from Ile27 (66) to Trp35 (74), characterized by several interruptions along the 100 ns. Through all simulations of restricted systems WT and H18L of Amber 99SB-ILDN force field, Arg30 (69) interacts with K29. The restricted system ΔH23Y24 presents this contact, however, with interruptions along the simulation time. Relatively to models constructed from homology modeling, contact with Ile27 (66) is also formed with longer interrupted intervals. In restricted system ΔH23Y24, these two residues contact with Lys29 continuously. The restricted Gromos 54a7 systems resulted in a more variable set of interactions. In the rest-unrestricted systems, the contacts are mainly formed with Ile27 (66), Asn28 (67), Arg30 (69), and Arg32 (71), with some differences between systems. In the ΔH23Y24 system of Amber 99SB-ILDN force field, only occasional interactions with two more residues of C3 are observed. For Gromos 54a7 force field, the profile is different, where two major interactions with Lys02 (41) and Arg03 (42) are observed, and occasionally contacts Arg32 (71) and three more residues are observed. In the H18L mutated structures of the Amber 99SB-ILDN force field, the interactions observed are similar with ΔH23Y24 system. The Gromos 54a7 force field system registered a distinct set of interactions with the C3 domain. In the WT systems of the Amber 99SB-ILDN force field, interactions with C3 domains and parallel residues of V3 are registered. In the Gromos 54a7 force field system a major interaction with Gln11 (50) is observed and no interactions with Ile27 (66) are registered.

The convergence of results between homology modeling and molecular dynamics concerning the interactions listed is confirmed. Relative to the 18-site of V3, a prevalence of interactions with Met15 (54), Val19 (58), and Phe20 (59) was seen, independent of mutations. In contrast, the removal of the aromatic moiety of this region seems to influence the access to this residue by the environment (e.g., the coreceptors). In the mutated systems, Ser16 (55) links non-covalently with Leu16, in opposition to the WT systems. The ΔH23Y24 mutants are characterized by many residues with which the residue His18 interacts momentarily.

## 4. Materials and Methods

### 4.1. Modelling the Structure of HIV-2ROD V3 Region

First, the sequence of HIV-2ROD gp160 envelope glycoprotein containing 858 amino acids was retrieved from UniProt, the Universal protein resource data bank (http://www.uniprot.org/), under the code P04577 [43]. Then, a search on the Protein Data Bank (http://www.rcsb.org/pdb/) was performed, based on this amino acid sequence, to obtain homologous proteins with the available 3D structure that could provide proper structural templates [44]. This procedure was replicated using the MOE to ensure the quality of the selected templates. The candidate lists were reduced by elimination of all hits presenting a low statistical significance (BLAST *E*-value greater than 0.01) or an alignment length shorter than 70% of the target sequences. Two crystallographic structures were identified as promising templates from HIV-1: HIV-1 YU2 envelope glycoprotein gp120 with V3 complexed with CD4 and tyrosine-sulfated 412d antibody (PDBID: 2QAD, resolution 3.3 Å) [30], HIV-1 JR-FL gp120 core envelope glycoprotein with V3 complexed with CD4 and the X5 antibody (PDBID: 2B4C, resolution 3.3 Å) [31].

Several models of the C2V3C3 envelope regions of HIV-2ROD were generated using Amber 99SB-ILDN99 forcefield [45] based on the crystallographic 3D structures of two templates of HIV-1 envelope glycoproteins. The quality of the models was analyzed using Ramachandran plots and the models with fewer outliers were selected and relaxed via energy minimization.

An additional model based on HIV-2ST gp125 envelope glycoprotein crystallographic structure (5CAY) was generated using the same procedure. This structure, a resolved HIV-2ST gp125 envelope glycoprotein bound to the first two domains of human soluble CD4 receptor (PDBID: 5CAY, resolution 3.0 Å) [27] was released during the construction of the models and selected to use in this protocol. Five residues from the N-terminus and seven residues from C-terminus were conserved in the HIV-2ST and HIV-1 V3 loops to allow for better alignment and superposition. The missing residues in HIV-2ST were derived from the 2QAD constructed minimized model. Then, a new homology model of the C2V3C3 portion of gp125 was generated. This new model was energy minimized (and optimized) and used as a template to generate a set of six new models with the following mutations and/or deletions at the V3 loop: H18L, ΔH23Y24, K29T, H18L + ΔH23Y24, H18L + K29T, and H18L + ΔH23Y24 + K29T. Secondary structure prediction was determined in Robetta (http://robetta.bakerlab.org/) [34] and PSIPRED (http://bioinf.cs.ucl.ac.uk/psipred/) [46,47] servers. To assess the overall quality of the models Ramachandran plots were analyzed, compared with the structure prediction. The models with fewer outliers and higher compliance with the structure prediction were selected and subjected to energy minimization using MOE. The statistics upon which the predictive model was based employed DSSP secondary structure assignments [48], which is the automatic secondary structure assignment algorithm built into MOE.

### 4.2. Molecular Dynamics of the Constructed Models

#### 4.2.1. System Setup

All molecular dynamics simulations were conducted using the GROMACS 2016.03 package. The starting coordinates were retrieved from the best homology models obtained for each individual system and were prepared using MOE 2015.10 software and further exported as PDB files.

To eliminate interactions with the respective periodic images and to promote periodic boundary conditions (PBC) in all dimensions, the molecules of each system were centered at a distance of 1.0 nm from the cubic box limit. The systems were solvated with water molecules using an SPC parameterized water molecules. The necessary number of chlorine counterions were added to neutralize the system. The initial topology and coordinates for each system were produced with GROMACS packages, using the all-atom Amber 99SB-ILDN united atom Gromos 54A7 force field [49,50,51,52].

#### 4.2.2. Simulation Details

Molecular dynamics simulations were performed on wild-type and six variants of the HIV-2ROD C2V3C3 region. Each solvated system was first subjected to 1000 steps steepest descent energy minimization to remove the unreasonable atomic contacts and stereochemical conflicts. Subsequently, a two sequential 1 ns NVT and NPT simulation step was carried away followed by a 100 ns MD simulation. The minimized systems were heated up to 300 K and then equilibrated under canonical NVT ensemble using the Velocity rescale weak coupling method [53]. The systems were then further optimized for 1000 ps in an isothermal-isobaric ensemble, NPT (300 K, 1 atm), using Parrinello-Rahman barostat [54] to an isotropic pressure coupling to a reference pressure of 1 bar, with a coupling time of 2 ps. As a default, the isothermal compressibility of water was defined at 4.5 × 10^−5^ bar^−1^. LINCS [55,56] algorithm was used to bond lengths constrain. A cut-off of 1.0 nm was used to compute the short-range electrostatic and van der Waals interactions. The Particle Mesh Ewald method [57,58] was used to calculate the long-range electrostatic interactions. The production runs were performed over 100 ns and under the same conditions. The configurations were saved each 100 ps for further analysis.

The analysis was performed using modules available in Gromacs 2016.3 package. The dynamic behavior and structural stability for all the systems were interpreted. All structural figures were prepared with MOE, PyMol molecular Graphics system, and VMD.

### 4.3. Key Interaction Determination

The PDB files of each system of the study were submitted using the PLIP algorithm in order to retrieve the intra-molecular interactions, through the python modulo PLIP [44]. This module analyses and visualizes protein-ligand interactions in PDB files. The PDB files were modified to consider the residue of interest as a ligand. It was possible to compare the interactions previously existent on the models from the homology modeling with the interactions existent along the molecular dynamics simulations. The full 100 ns trajectory was divided into 1000 snapshots to analyze each one. The interactions herein considered involving: H-bonds, hydrophobic contacts, π-stacking, water bridges, halogen-bonds, and salt bridges. The parameters used by the PLIP module can be accessed in the file config.py [44].

## 5. Conclusions

In the absence of a crystallographic structure of HIV-2 envelope gp125 comprising variable domains, computer-aided modeling is crucial to identify structural features in the variable V3 region that correlate with HIV-2 co-receptor use, tropism, and susceptibility to antibody neutralization. A 3D structure of the C2V3C3 domain of HIV-2ROD gp125 was generated by homology modeling. HIV-2ROD is an X4 T-cell adapted isolate naturally resistant to antibody neutralization. To disclose the importance of the main structural features and for comparison with experimental results, 3D-models of six V3 mutants were also generated (H18L, ΔH23Y24, K29T, H18L + ΔH23Y24, H18L + K29T and H18L + ΔH23Y24 + K29T). These mutations in V3 revealed selective impact in co-receptor use [24].

The H18L variant replaces a basic residue with a hydrophobic one leading to the loss of an aromatic moiety and abrogating any π-π interactions in this position. HIV-2 cell entry comprises the interaction of the envelope glycoprotein gp125 with CD4, exposure of gp125 V3 loop, and the binding to chemokine receptors CCR5 or CXCR4.

The modification of the nature of the residue on this position could interfere with the type of interaction that can be performed with the environment, namely specific π-π stacking and π-cation interactions can be broken by this mutation. The H18L variant replaces a basic residue with a hydrophobic residue and leads to the loss of the aromatic moiety which abrogates any possibility of establishment of π-π or π-cation interactions within the V3 environment or eventually with CCR5 residues Ile12, Tyr15, Tyr108, Phe109, Phe112, Phe172, Tyr187, Ile198, Trp248, and Tyr251 or with CXCR4 Leu14 Trp20, Tyr21, Lys32, Phe189, Tyr190, and Val196 [59,60]. The modifications that the mutation lead in terms of interactions of the whole residue can interfere with the loop. The complete crystal structure of the co-receptor with the loop could accomplish this analysis. Future molecular work can be developed and the data that we give here could help in those studies.

In the wild-type (WT) HIV-2ROD V3, His18 forms H-bonds with Met15 and Phe20. His23 and Tyr24 are placed on a β sheet. The β sheet content present in WT V3 totalizes 23% of the total loop. His23 interacts with Gly25 and Gly11. Tyr24 can establish π-π interactions and exposition of its hydroxyl group could promote other interactions with the environment (i.e., cell co-receptors and antibodies). The deletion of these two residues, along with the H18L substitution, results in the elimination of the parallel β sheets and a major loss of the aromatic system. Experimentally, such changes were associated with X4-to-R5 coreceptor switch and, in preliminary studies, higher sensitivity to antibody neutralization. The substitution of Lys29 by a threonine reduces the charge of V3 and leads to loss of the interactions with Ile27. This did not cause a change in co-receptor use but, in preliminary studies, it was associated with higher resistance to antibody neutralization and acquisition of macrophage tropism. The production of MD simulations helps to disclose the dynamic behavior of the variants studied and it will be further studied to lead to a better final model of this glycoprotein.

These new insights into the structure-function relationship of HIV-2 V3 and surrounding regions will help in the design of better models and the design of new small molecules capable of inhibiting the attachment and binding of HIV with host cells.

## Figures and Tables

**Table 1:** Presentation of the systems studied. Mutations and/or deletions are identified (blue residues).

V3 Systems	V3 Sequence							
ROD WT	1	5	10	15	20	25	30	35
	CKRPGNKTVKQIMLMSGHVFHSHYQPINKRPRQAWC							
H18L	CKRPGNKTVKQIMLMSGLVFHSHYQPINKRPRQAWC							
k29T	CKRPGNKTVKQIMLMSGHVFHSHYQPINTRPRQAWC							
ΔH23Y24	CKRPGNKTVKQIMLMSGHVFHSHYQPINKRPRQAWC							
H18L_K29T	CKRPGNKTVKQIMLMSGLVFHSHYQPINTRPRQAWC							
H18L_ΔH23Y24	CKRPGNKTVKQIMLMSGLVFHSHYQPINKRPRQAWC							
H18L_K29T_ΔH23Y24	CKRPGNKTVKQIMLMSGLVFHSHYQPINTRPRQAWC							

**Table 2:** HIV-1 and HIV-2 V3 sequences used to generate the HIV-2 gp125 V3 structural models.

Strain of the Virus (PDB Identifier)	V3 Sequence	Res. (Å)
HIV-1YU2 (2QAD)	CTRPNNNTRKSINIQRGPGRALYTTGEIIGDIRQAHC	3.3
HIV-1 JR-FL (2B4C)	CTRPNQNTRKSIHIQRGPGRAFYTTGEIIGDIRQAHC	3.5
HIV-2ST (5CAY)	CKRPGNKTVVPITLMSGLVFHSHYQPINRRPRQAWC	3.0
HIV-2ROD	CKRPGNKTVKQIMLMSGHVFHSHYQPINKRPRQAWC	--

**Table 3:** Structural prediction for HIV-2 ROD V3 region. The α Helix is defined in dark gray and β Sheet is defined in the pattern cells.

		V3 Sequence																																		
Residues	C	K	R	P	G	N	K	T	V	K	Q	I	M	L	M	S	G	H	V	F	H	S	H	Y	Q	P	I	N	K	R	P	R	Q	A	W	C
**PSIPRED**																																				
**Robetta**																																				
**Model**																																				
		β Sheet								α Helix																										

**Table 4:** Solvent-accessible surface area (SASA), charge, length, and width of ROD WT and mutant V3 loops.

V3 Region	RMSD	SASA (Å^2^)	Δ (SASA)	Charge	Length (Å)	Width (Å)
ROD WT	0	3852.3	0	+7	47.50	5.30
H18L	0.096	3853.1	0.8	+7	47.67	5.34
k29T	0.144	3834.4	−17.9	+6	47.67	5.34
ΔH23Y24	2.812	3790.8	−61.5	+7	47.67	6.17
H18L_K29T	0.150	3821.9	−30.4	+6	47.68	5.34
H18L_ΔH23Y24	1.107	3778.5	−73.8	+7	47.50	6.13
H18L_K29T_ΔH23Y24	1.128	3760.5	−91.8	+6	47.50	6.13

**Table 5:** Resume of all the systems used in Molecular dynamics.

V3 Region	Amber 99SB-ILDN			Gromos 54a7		
ROD WT	C2 and C3 unrest	C2 and C3 rest	C2 and C3 unrest after restriction	C2 and C3 unrest	C2 and C3 rest	C2 and C3 unrest after restriction
H18L	C2 and C3 unrest	C2 and C3 rest	C2 and C3 unrest after restriction	C2 and C3 unrest	C2 and C3 rest	C2 and C3 unrest after restriction
ΔH23Y24	C2 and C3 unrest	C2 and C3 rest	C2 and C3 unrest after restriction	C2 and C3 unrest	C2 and C3 rest	C2 and C3 unrest after restriction

**Table 6:** Mean potential energy values and standard deviation.

	Amber 99SB-ILDN			Gromos 54a7		
Variant	Unrest	Rest	Unrest after Restriction	Unrest	Rest	Unrest after Restriction
ROD WT	−513,539.48 ± 1087.30	−512,927.52 ± 1082.42	−513,472.38 ± 1039.24	−867,141.25 ± 1358.80	−866,617.64 ± 1326.67	−867,171.99 ± 1321.13
H18L	−571,028.67 ± 1125.78	−570,290.46 ± 1123.11	−570,940.25 ± 1126.62	−1,461,028.35 ± 1629.22	−1,460,455.02 ± 1782.04	−1,461,030.00 ± 1811,57
ΔH23Y24	−1,124,328.83 ± 1491.94	−1,123,663.29 ± 1625.08	−1,124,215.34 ± 1613.15	−602,249.39 ± 1112.07	−601,613.97 ± 1137.73	−602,404.45 ± 1090.76

**Table 7:** Mean RMSD values and standard deviation after the 40 ns.

	Amber 99SB-ILDN			Gromos 54a7		
Variant	Unrest	Rest	Unrest after Restriction	Unrest	Rest	Unrest after Restriction
ROD WT	1.124 ± 0.015	0.849 ± 0.045	0.951 ± 0.087	0.966 ± 0.046	0.705 ± 0.018	0.789 ± 0.033
H18L	0.852 ± 0.132	0.393 ± 0.125	0.966 ± 0.149	0.911 ± 0.197	1.224 ± 0.065	0.729 ± 0.069
ΔH23Y24	1.026 ± 0.041	0.542 ± 0.052	0.691 ± 0.041	0.870 ± 0.032	0.521 ± 0.091	0.754 ± 0.058

## Supplementary Materials

The following are available online at https://www.mdpi.com/1422-0067/22/4/1948/s1.
