# Supplementary material for: Computational Modulation of the V3 Region of Glycoprotein gp125 of HIV-2
Source: Int J Mol Sci. 2021 Feb 16;22(4):1948. doi: 10.3390/ijms22041948 (PMC7920276; doi:10.3390/ijms22041948)
Supplement: Supplementary file 1 [file ijms-22-01948-s001.pdf]

Supplementary Information

Computational modulation of the V3 region of glycoprotein gp125 of HIV-2

Patrícia A. Serra [1], Nuno Taveira [1,2,*] and Rita C. Guedes [1,*]

[1] Instituto de Investigação do Medicamento (iMed.ULisboa), Faculdade de Farmácia, Universidade de Lisboa, Avenida Professor Gama Pinto, Lisboa, Portugal
[2] Centro de Investigação Interdisciplinar Egas Moniz (CiiEM), Instituto Universitário Egas Moniz, Monte de Caparica, Portugal
* Correspondence: ntaveira@ff.ulisboa.pt; rguedes@ff.ulisboa.pt

Supplementary information 1 | validation plots of the wt model 2

Supplementary information 2 | plif plots of c2v3c3 3

Supplementary information 1| Validation plots of the WT model

Ramachandran Plots and ERRAT plot:

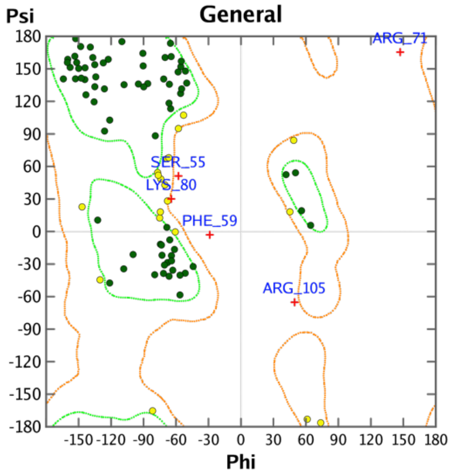

Figure 1. WT Ramachandran plot

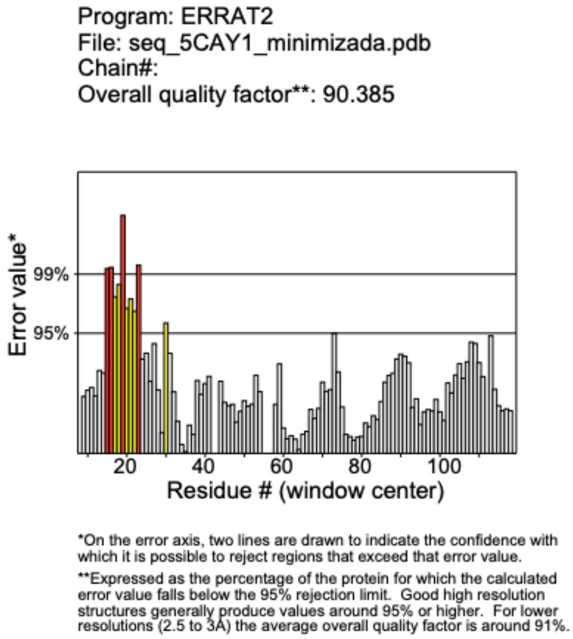

Figure 2. WT Errat validation plot

Supplementary information 2| PLIF plots of C2V3C3

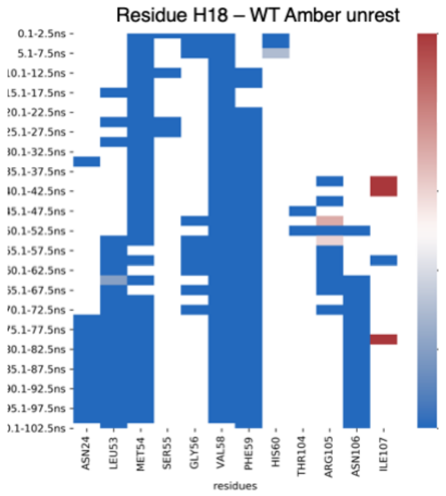

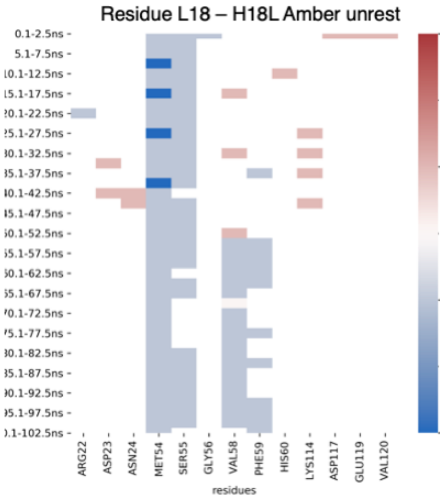

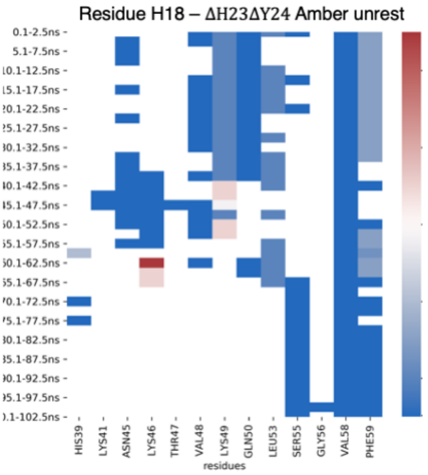

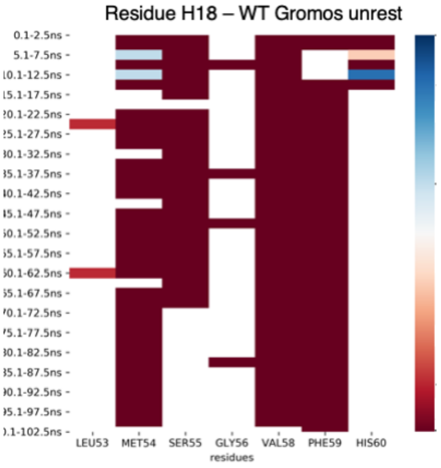

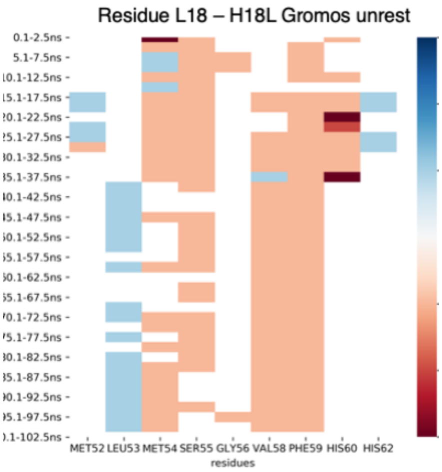

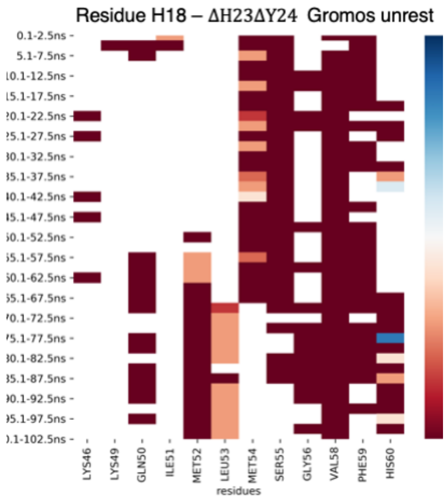

Residue H18 – WT Amber rest-unrest

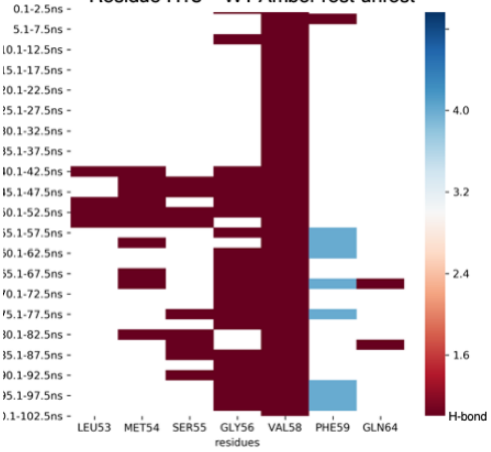

Residue L18 - H18L Amber rest-unrest

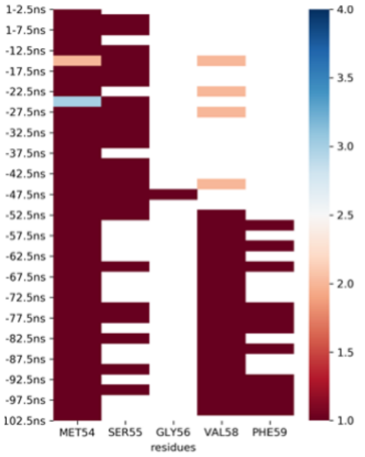

Residue H18 – ΔH23ΔY24 Amber rest-unrest

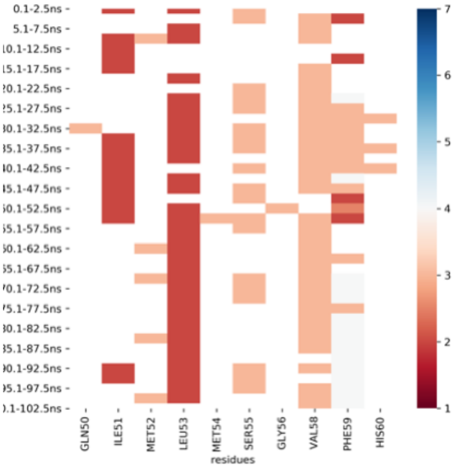

Residue H18 – WT Gromos rest-unrest

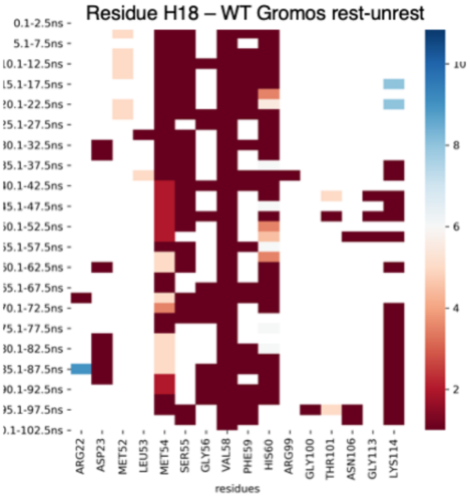

Residue L18 - H18L Gromos rest-unrest

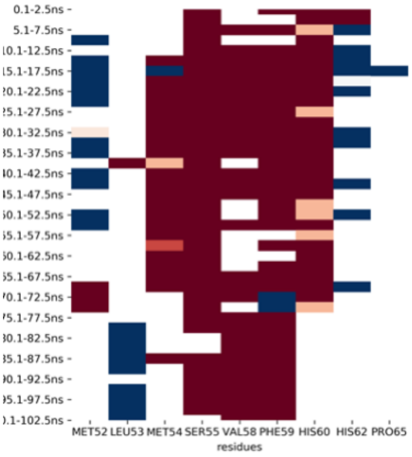

Residue H18 – ΔH23ΔY24 Gromos rest-unrest

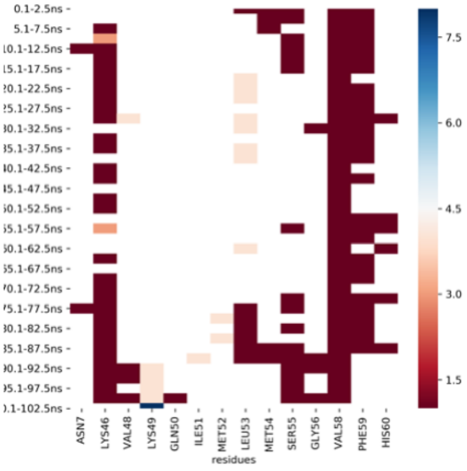

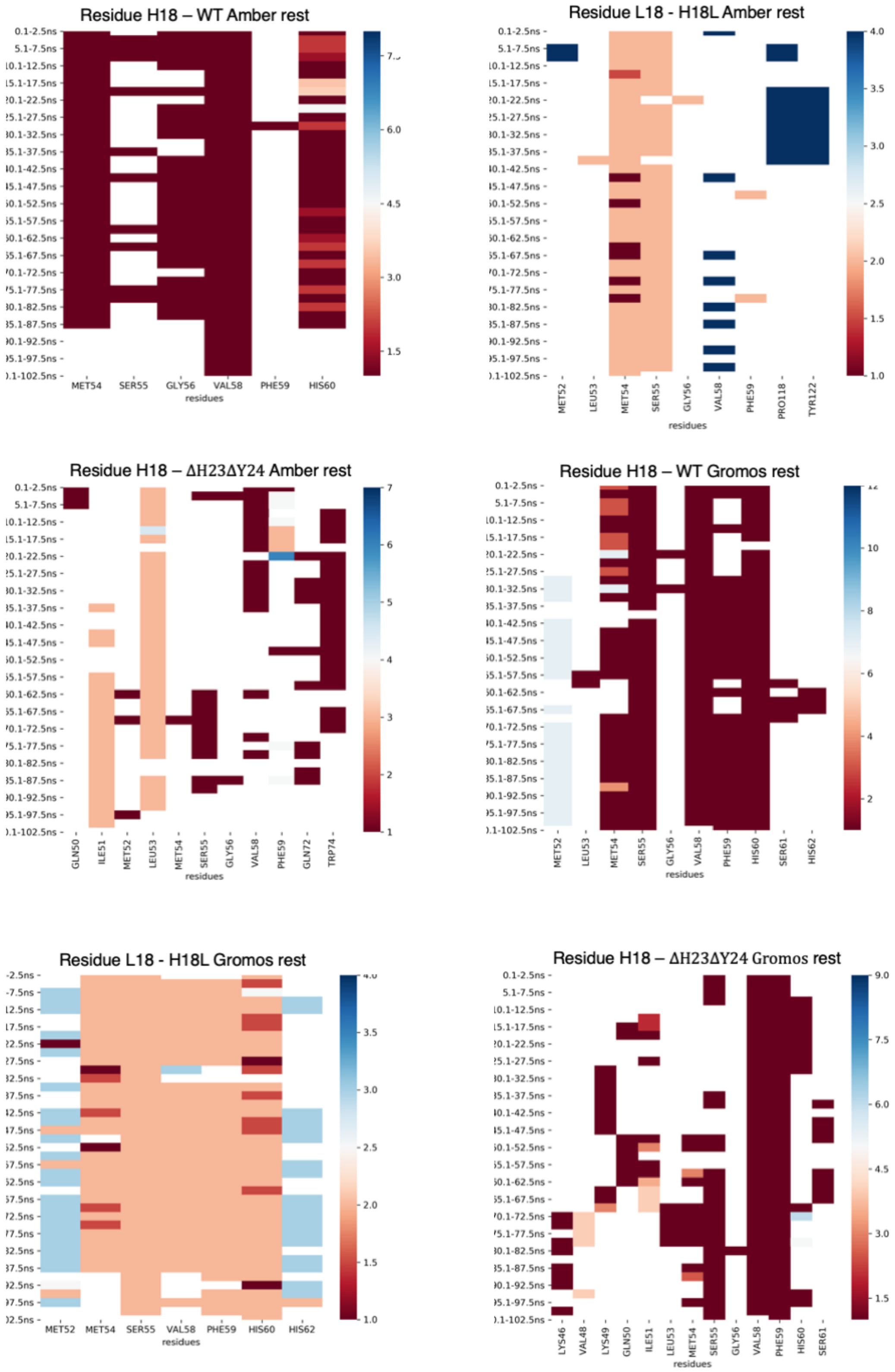

Residue H18 – WT Amber rest
MET54 SER55 GLY56 VAL58 PHE59 HIS60
residues
Residue L18 - H18L Amber rest
MET52 LEU53 MET54 SER55 GLY56 VAL58 PHE59 PRO118 TYR122
residues
Residue H18 – ΔH23ΔY24 Amber rest
GLN50 ILE51 MET52 LEU53 MET54 SER55 GLY56 VAL58 PHE59 GLN72 TRP74
residues
Residue H18 – WT Gromos rest
MET52 LEU53 MET54 SER55 GLY56 VAL58 PHE59 HIS60 SER61 HIS62
residues
Residue L18 - H18L Gromos rest
MET52 MET54 SER55 VAL58 PHE59 HIS60 HIS62
residues
Residue H18 – ΔH23ΔY24 Gromos rest
LYS46 VAL48 LYS49 GLN50 ILE51 LEU53 MET54 SER55 GLY56 VAL58 PHE59 HIS60 SER61
residues

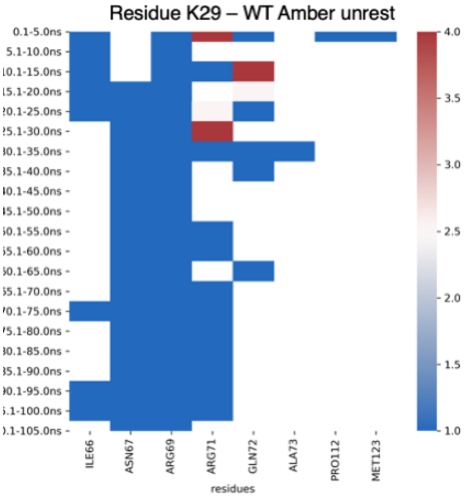
Residue K29 – WT Amber unrest
ILE66
ASN67
ARG69
ARG71
GLN72
ALA73
PRO112
MET123
residues

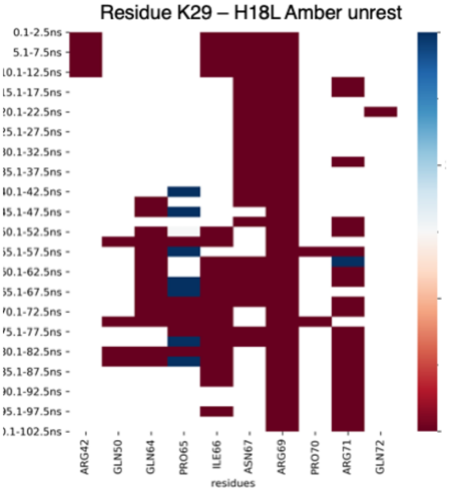
Residue K29 – H18L Amber unrest
ARG42
GLN50
GLN64
PRO65
ILE66
ASN67
ARG69
PRO70
ARG71
GLN72
residues

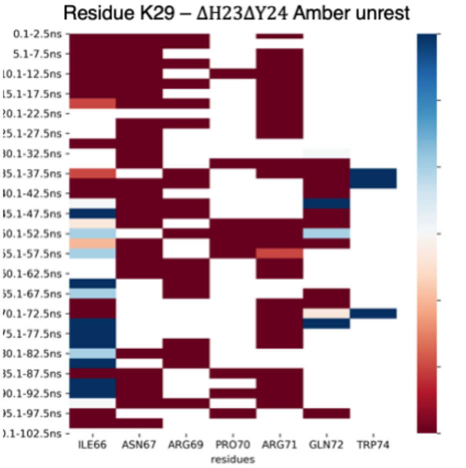
Residue K29 – ΔH23ΔY24 Amber unrest
ILE66
ASN67
ARG69
PRO70
ARG71
GLN72
TRP74
residues

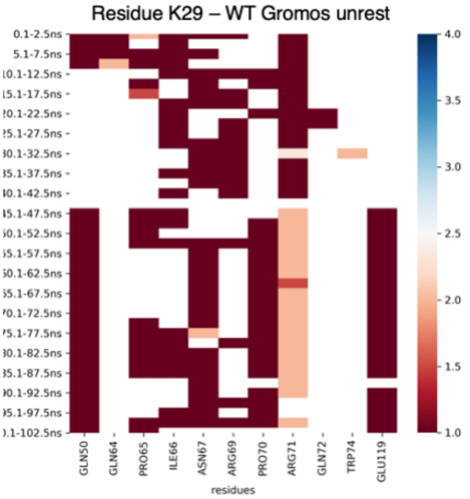
Residue K29 – WT Gromos unrest
GLN50
GLN64
PRO65
ILE66
ASN67
ARG69
PRO70
ARG71
GLN72
TRP74
GLU119
residues

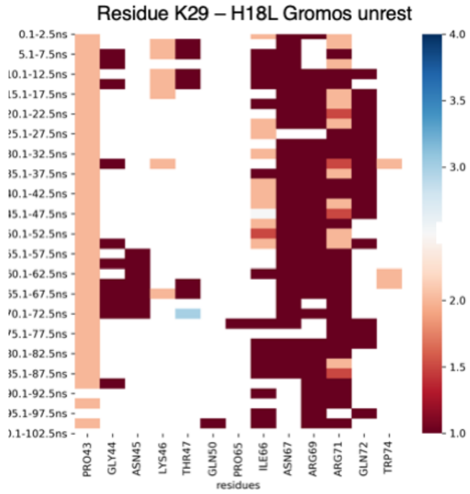
Residue K29 – H18L Gromos unrest
PRO43
GLY44
ASN45
LYS46
THR47
GLN50
PRO65
ILE66
ASN67
ARG69
ARG71
GLN72
TRP74
residues

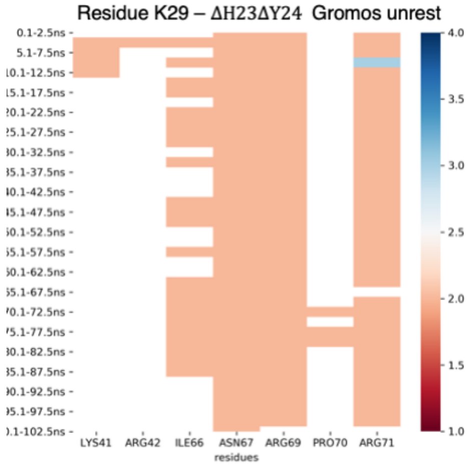
Residue K29 – ΔH23ΔY24 Gromos unrest
LYS41
ARG42
ILE66
ASN67
ARG69
PRO70
ARG71
residues

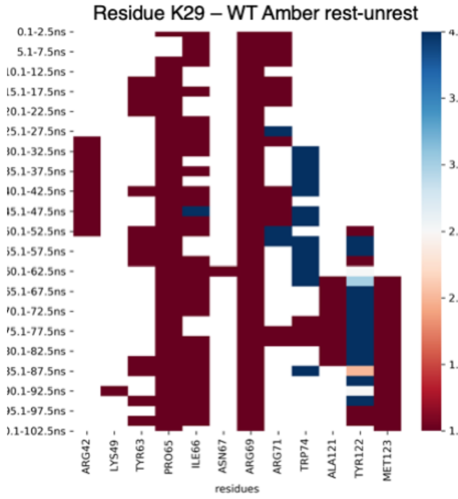
Residue K29 – WT Amber rest-unrest
ARG42 LYS49 TYR63 PRO65 ILE66 ASN67 ARG69 ARG71 TRP74 ALA121 TYR122 MET123
residues

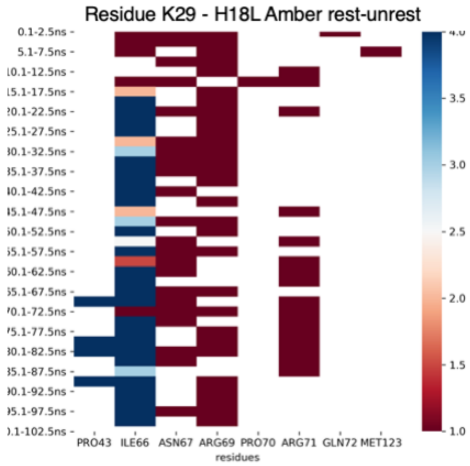
Residue K29 - H18L Amber rest-unrest
PRO43 ILE66 ASN67 ARG69 PRO70 ARG71 GLN72 MET123
residues

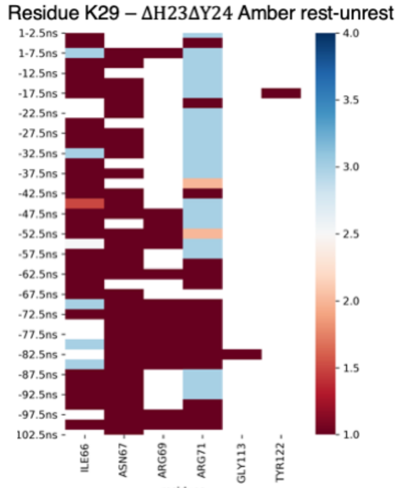
Residue K29 – ΔH23ΔY24 Amber rest-unrest
ILE66 ASN67 ARG69 ARG71 GLY113 TYR122
residues

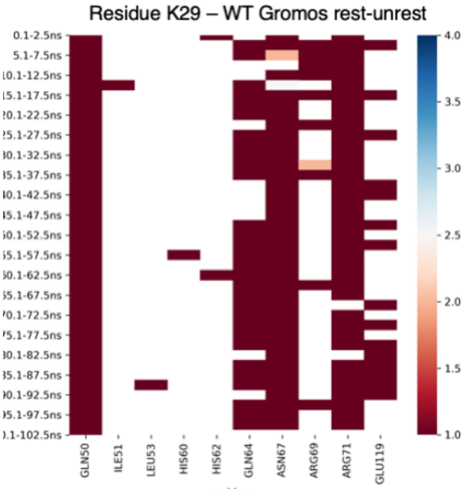
Residue K29 – WT Gromos rest-unrest
GLN50 ILE51 LEU53 HIS60 HIS62 GLN64 ASN67 ARG69 ARG71 GLU119
residues

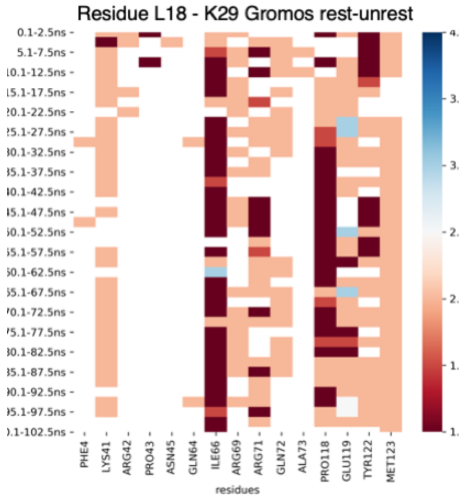
Residue L18 - K29 Gromos rest-unrest
PHE4 LYS41 ARG42 PRO43 ASN45 GLN64 ILE66 ARG69 ARG71 GLN72 ALA73 PRO118 GLU119 TYR122 MET123
residues

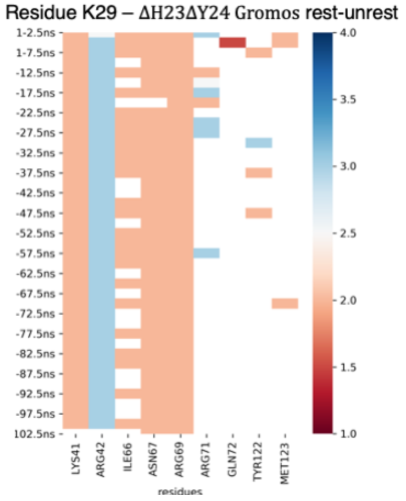
Residue K29 – ΔH23ΔY24 Gromos rest-unrest
LYS41 ARG42 ILE66 ASN67 ARG69 ARG71 GLN72 TYR122 MET123
residues

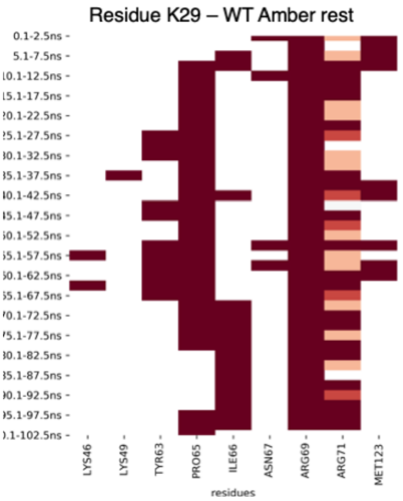
Residue K29 – WT Amber rest
LYS46
LYS49
TYR63
PRO65
ILE66
ASN67
ARG69
ARG71
MET123
residues

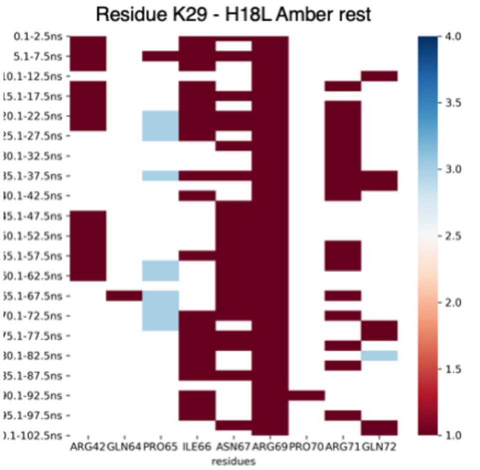
Residue K29 - H18L Amber rest
ARG42
GLN64
PRO65
ILE66
ASN67
ARG69
PRO70
ARG71
GLN72
residues

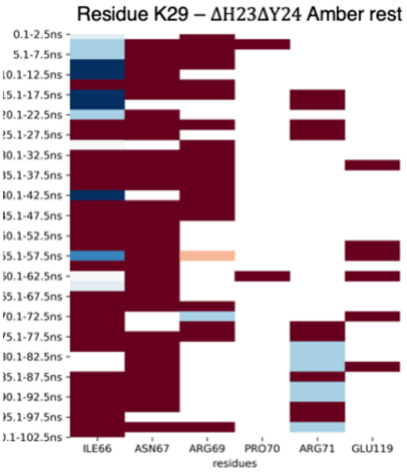
Residue K29 – ΔH23ΔY24 Amber rest
ILE66
ASN67
ARG69
PRO70
ARG71
GLU119
residues

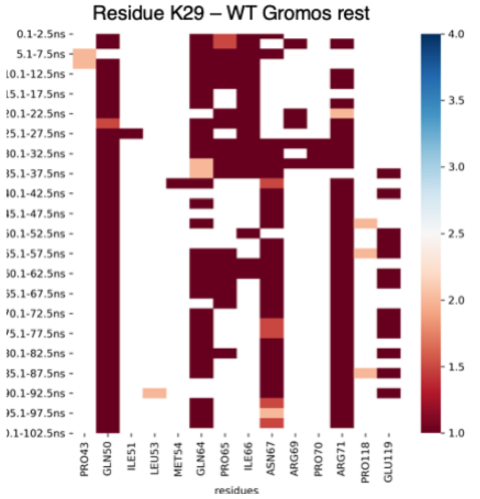
Residue K29 – WT Gromos rest
PRO43
GLN50
ILE51
LEU53
MET54
GLN64
PRO65
ILE66
ASN67
ARG69
PRO70
ARG71
PRO118
GLU119
residues

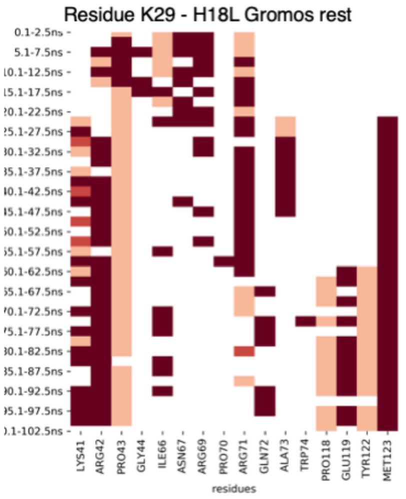
Residue K29 - H18L Gromos rest
LYS41
ARG42
PRO43
GLY44
ILE66
ASN67
ARG69
PRO70
ARG71
GLN72
ALA73
TRP74
PRO118
GLU119
TYR122
MET123
residues

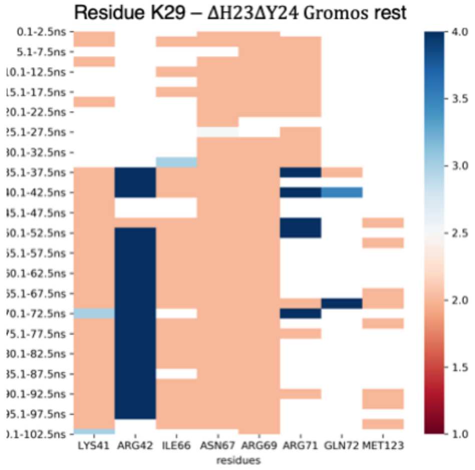
Residue K29 – ΔH23ΔY24 Gromos rest
LYS41
ARG42
ILE66
ASN67
ARG69
ARG71
GLN72
MET123
residues
